# Mitral Valve Prolapse and Sudden Cardiac Death—A Puzzle with Missing Pieces: Review of the Literature and Case Report

**DOI:** 10.3390/medsci13030185

**Published:** 2025-09-10

**Authors:** Diana Roxana Opris, Marius Mihai Harpa, David-Emanuel Anitei, Paul Calburean, Roxana Rudzik

**Affiliations:** 1 Doctoral School, George Emil Palade University of Medicine, Pharmacy, Science and Technology of Targu Mures, 540142 Targu Mures, Romania; dianaroxana.opris@yahoo.com; 2 Internal Medicine V, George Emil Palade University of Medicine, Pharmacy, Science, and Technology of Targu Mures, 540142 Targu Mures, Romania; 3 Department of Cardiology, Emergency Institute for Cardiovascular Diseases and Transplantation Targu Mures, 540136 Targu Mures, Romania; roxana.rudzik@ibcvt.ro; 4 Department of Surgery IV, George Emil Palade University of Medicine, Pharmacy, Science and Technology of Targu Mures, 540142 Targu Mures, Romania; 5 Department of Cardiovascular Surgery, Emergency Institute for Cardiovascular Diseases and Transplantation Targu Mures, 540136 Targu Mures, Romania; anitei_emanuel@yahoo.com; 6 Department of Biostatistics and Medical Informatics, George Emil Palade University of Medicine, Pharmacy, Science and Technology of Targu Mures, 540142 Targu Mures, Romania; calbureanpaul@gmail.com

**Keywords:** mitral regurgitation, annulus disjunction, sudden cardiac death, ventricular arrhythmias, minimally invasive surgery

## Abstract

**Background:** Mitral valve prolapse is a common valvular heart disorder, usually associated with a benign prognosis in the absence of significant mitral regurgitation. However, a subset of patients is at increased risk for complex ventricular arrhythmias and sudden cardiac death. Identifying these high-risk individuals remains a major clinical challenge. **Case Summary:** We present the case of a 71-year-old female patient with recurrent syncopal episodes, a strong family history of sudden cardiac death, and complex ventricular ectopy. Multimodality imaging revealed bileaflet mitral valve prolapse, severe mitral regurgitation, mitral annular disjunction, and the Pickelhaube sign, with no evidence of myocardial fibrosis on cardiac magnetic resonance imaging. The patient underwent minimally invasive mitral valve repair and received an implantable cardioverter-defibrillator for primary prevention of sudden cardiac death. Follow-up revealed significant reverse cardiac remodeling, marked reduction in arrhythmic burden, and restoration of mitral valve function. Family screening identified mitral annular disjunction in both of her daughters, who were asymptomatic and without arrhythmias. **Discussion:** Mitral annular disjunction has emerged as a potentially arrhythmogenic substrate, especially in patients with familial clustering, raising the possibility of a genetic predisposition. Risk stratification remains difficult, as no individual clinical, electrocardiographic, or imaging marker has demonstrated consistent predictive value. Surgical correction of mitral valve prolapse with associated mitral annular disjunction may lead to a reduction in arrhythmic risk and promote favorable structural remodeling. **Conclusions:** This case-based review emphasizes the importance of advanced imaging techniques in the identification and management of high-risk mitral valve prolapse phenotypes. Early surgical intervention and close arrhythmic surveillance may improve outcomes, although further research is necessary to define risk assessment tools and explore the genetic background of arrhythmogenic mitral valve disease.

## 1. Introduction

Mitral valve prolapse is a frequent valvular heart disease [1], well characterized by echocardiography. In the absence of mitral regurgitation and its impact on left ventricular function, it is generally associated with a benign clinical course [2,3]. However, a small and incompletely characterized subset of patients is at increased risk of developing ventricular arrhythmias and sudden cardiac death. Several clinical, electrocardiographic, and imaging features have been associated with malignant arrhythmic events in such patients [4]. This wide clinical spectrum and the potential for sudden cardiac death highlight the urgent need for improved risk stratification strategies. Advanced cardiac imaging plays a pivotal role not only in the early detection of arrhythmogenic mitral valve prolapse, mitral annular disjunction, and associated structural abnormalities but also in the longitudinal monitoring of disease progression. Furthermore, careful clinical and imaging follow-up can reveal reverse cardiac remodeling in patients who undergo timely surgical intervention, emphasizing the role of early mitral valve repair guided by imaging findings [5]. This case report presents a patient with arrhythmogenic mitral valve disease and discusses the specific characteristics of high-risk groups and therapeutic approaches.

## 2. Case Presentation

A 71-year-old female patient was referred for cardiologic evaluation following a syncopal episode complicated by a nasal bone fracture requiring surgical correction. Clinical examination revealed a holosystolic murmur, best heard at the apex and radiating to the axilla. The patient was subsequently referred for further comprehensive cardiologic assessment. Prior to admission, she experienced three additional syncopal episodes and several presyncopal events. She also reported episodes of rapid, irregular palpitations lasting up to 30 s, associated with light-headedness and dyspnoea, with sudden onset and offset, all occurring within the preceding year.

A neurological cause had previously been ruled out through carotid Doppler ultrasound, multiple electroencephalograms, and cranial computed tomography. The patient was not taking any medications and had no other known medical conditions. Of note, two family members (her father and brother) had died suddenly from presumed cardiac causes around the age of 50.

**Figure 1:**
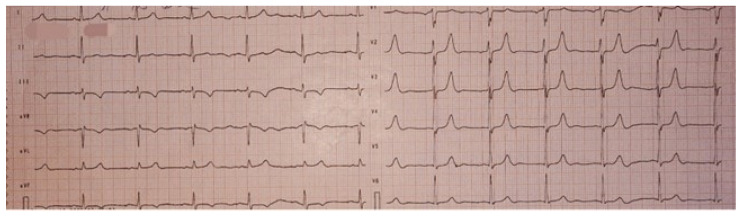
Twelve-lead electrocardiogram (ECG) showing sinus rhythm, without evidence of conduction abnormalities, with T wave inversion in leads DIII and aVF, and a normal QTc.

On physical examination, the holosystolic murmur was confirmed as significant, with no other remarkable findings. A 12-lead electrocardiogram showed sinus rhythm and T-wave inversions in leads DIII and aVF, without evidence of conduction abnormalities or QTc interval prolongation (Figure 1).

No pathological findings were identified on the complete blood count. Renal, hepatic, electrolyte, and thyroid function tests were within normal limits. The B-type natriuretic peptide level was 500 pg/mL.

**Figure 2:**
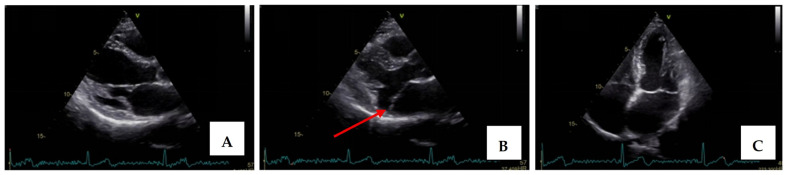
Mitral valve prolapse with MAD on transthoracic echocardiography (TTE). (**A**)—TTE parasternal long-axis view in end-diastole displaying excessive leaflet length and thickness, (**B**)—TTE parasternal long-axis view in end-systole displaying mitral annular disjunction-MAD of 10 mm length (red arrow), and (**C**)—apical 4-chamber view displaying a displacement of both leaflets > 2 mm above the plane of the annulus.

**Figure 3:**
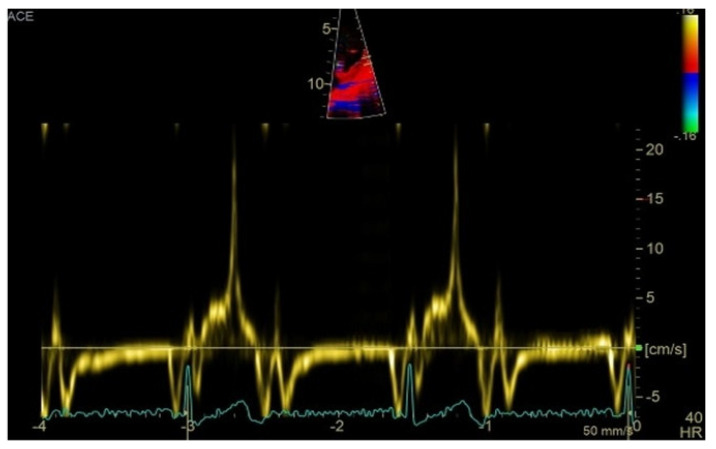
High-velocity spike (21 cm/s) between mid-systole to late-systole using tissue Doppler imaging on the lateral mitral annulus. Known as the Pickelhaube sign, this sharp annular spike resembles the spike that adorns the Pickelhaube helmet, historically worn by the German military. It has been proposed as a marker of arrhythmogenic mitral valve prolapse.

Transthoracic echocardiography revealed myxomatous degeneration of the mitral valve with bileaflet redundancy, increased leaflet length and thickness, and prolapse of both leaflets. Notably, a mitral annular disjunction (MAD) measuring 10 mm was observed in the parasternal long-axis view at end-systole (Figure 2A–C). Additionally, systolic “curling motion” of the posterolateral wall of the left ventricle was present, and the Pickelhaube sign was documented (Figure 3).

**Figure 4:**
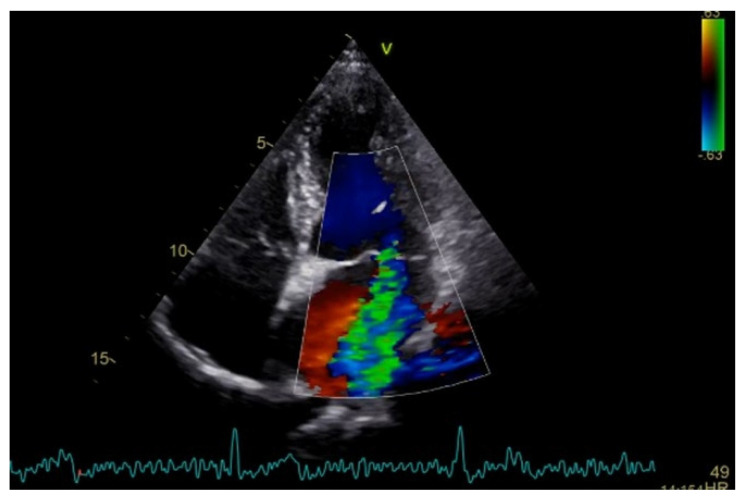
Apical four chamber view showing severe mitral regurgitation with left atrial dilatation.

**Figure 5:**
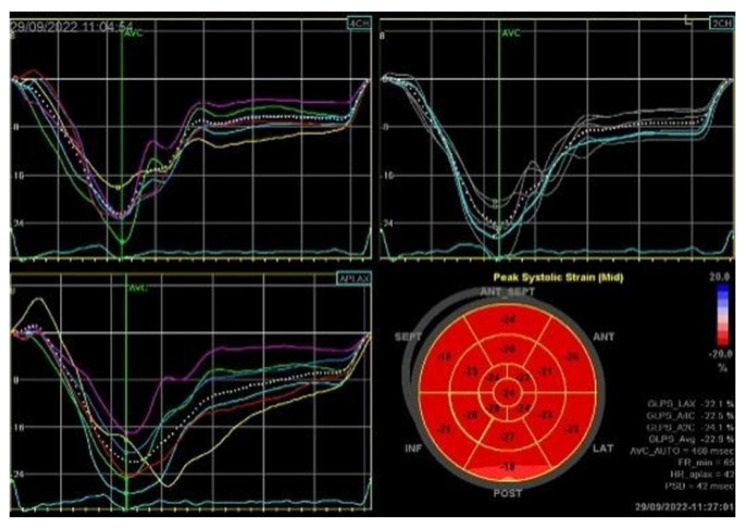
2D speckle-tracking echocardiography used as a tool for assessing left ventricular systolic performance (GLS average −22.9%), with some degree of dispersion in longitudinal strain peaks.

Severe mitral regurgitation was identified, manifested by an eccentric regurgitant jet and associated left atrial dilatation (Figure 4). Moderate tricuspid regurgitation with right atrial dilatation was also present. Both the left and right ventricles were of normal size and function. Global longitudinal strain was preserved for both ventricles, with an average of −22.9% for the left ventricle and −22.0% for the right ventricle, although a mild degree of dispersion in longitudinal strain peaks was observed (Figure 5).

**Figure 6:**
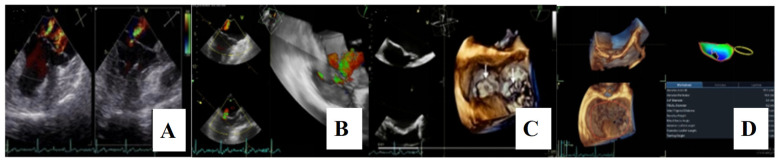
(**A**,**B**)—Transoesophageal echocardiography (TEE) showing severe mitral regurgitation. (**C**,**D**)—TEE 3D view, displaying bileaflet prolapse and mitral annular enlargement. Mitral valve apparatus geometry and mitral valve dynamics were evaluated using EchoPAC software version BT12.

For a comprehensive morphological and functional evaluation of the mitral valve, transesophageal echocardiography was performed. Three-dimensional imaging provided an “en face” view of the mitral leaflets, which confirmed the previous diagnosis of bileaflet mitral valve prolapse involving scallops A1, A2, A3 and P1, P2, P3. Severe mitral regurgitation and moderate tricuspid regurgitation were also documented (Figure 6A–D).

**Figure 7:**
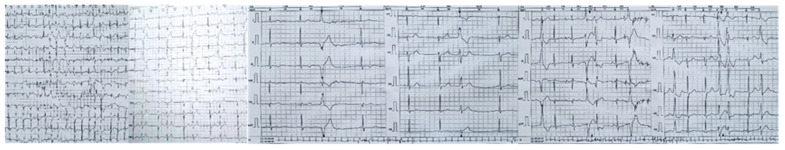
Twenty-four-hour Holter monitor showing complex ventricular ectopy likely from the postero-medial papillary muscle, antero-lateral papillary muscle, and from the mitral annulus. This localization, determined using established ECG criteria and algorithms, supports the concept of papillary muscle–mediated arrhythmias in arrhythmogenic MVP. Short-coupled premature ventricular contraction—PVCs, bigeminy, and ventricular couplets, as observed in this patient, are recognized triggers for ventricular tachyarrhythmias and may contribute to sudden cardiac death risk in this population.

Twenty-four-hour Holter monitoring revealed frequent polymorphic premature ventricular contractions, with a total ventricular ectopic burden of 16% over 24 h. Based on established electrocardiographic criteria and localization algorithms, the likely origin of these ventricular arrhythmias was traced to the posterior and anterior papillary muscles, as well as to the mitral annulus. Some of the ectopic beats exhibited short coupling intervals. Additionally, bigeminy, ventricular couplets, and episodes of non-sustained ventricular tachycardia were observed (Figure 7).

Coronary angiography demonstrated no evidence of obstructive coronary artery disease. An electrophysiological study was recommended to further evaluate the arrhythmic substrate and assess the inducibility of ventricular arrhythmias; however, the patient declined the procedure.

**Figure 8:**
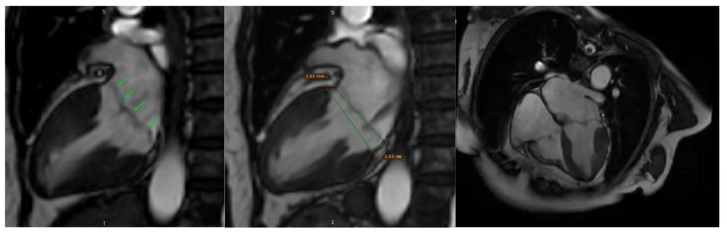
Mitral valve prolapse and mitral annulus disjunction (MAD) on cardiac magnetic resonance (CMR). Green arrows indicate myxomatous valve changes and mitral valve prolapse. Green lines illustrate mitral annular disjunction (MAD), represented as the distance between the two lines: the first drawn at the level of the mitral annulus and the second at the level of the ventricular myocardium.

Additionally, contrast-enhanced cardiac magnetic resonance imaging (MRI) confirmed the previously described findings, including bileaflet mitral valve prolapse with leaflet redundancy, severe mitral regurgitation, and mitral annular disjunction. The left ventricle was of normal size and function, and there was no late gadolinium enhancement (LGE), indicating the absence of myocardial fibrosis (Figure 8).

**Figure 9:**
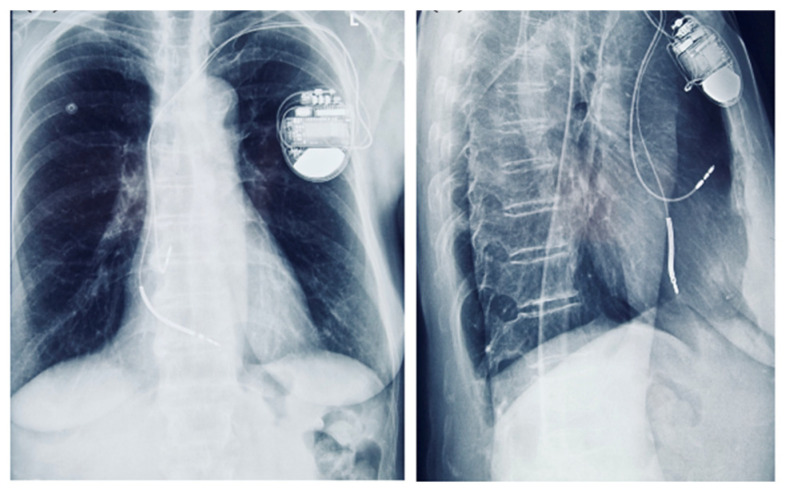
Chest radiograph showing a dual-chamber implantable cardioverter-defibrillator (ICD) in anteroposterior and lateral views.

Although the patient had no sustained VT and no LGE on CMR, implantable cardioverter-defibrillator (ICD) implantation was justified by the presence of multiple high-risk features. Following Heart Team discussion, an ICD was implanted for primary prevention of sudden cardiac death, in the context of arrhythmogenic mitral valve prolapse (AMVP) with high-risk features (Figure 9). Moreover, given the additional need for beta-blocker therapy and the patient’s baseline bradycardia, a dual-chamber ICD was selected to provide both arrhythmic protection and pacing support. The presence of bradycardia, together with the requirement for antiarrhythmic medication, represented an additional factor influencing the decision for ICD implantation.

To address the severe mitral regurgitation, the patient underwent three-dimensional video-assisted thoracoscopic mitral valvuloplasty and annuloplasty via a minimally invasive approach. The procedure included implantation of neochordae loops—four to the anterior leaflet and five to the posterior leaflet.

**Figure 10:**
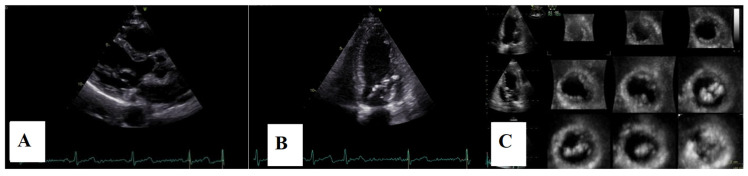
(**A**)—Postoperative TTE parasternal long axis view. (**B**)—apical four-chamber view. (**C**)—3D echocardiography, 12 slices, displaying hyperecogenic mitral annulus, hyperecogenic mitral leaflets without billowing, and two groups of hyperechoic chordae inserted on the papillary muscle level.

Postoperative transthoracic echocardiographic evaluation showed a left ventricle of normal size and function, a hyperechogenic mitral annulus, and hyperechogenic mitral leaflets without billowing, with only minor residual regurgitation. Additionally, two distinct groups of hyperechoic chordae were visualized at the level of the papillary muscles (Figure 10).

Postoperative follow-up was performed dynamically at 3, 6, and 12 months, including telemetry, Holter electrocardiography, laboratory testing (complete blood count and N-terminal pro–B-type natriuretic peptide), as well as bi- and three-dimensional echocardiographic evaluation. Device interrogation revealed no delivered therapies, no recorded arrhythmic episodes, and a high percentage of right atrial pacing. Serial laboratory analysis demonstrated a progressive decrease in leukocyte count (from 9800 to 8700 and then 5300/mm^3^) and neutrophils, along with a reduction in N-terminal pro–B-type natriuretic peptide levels, which normalized by the 6-month follow-up.

Holter monitoring showed a significant decrease in arrhythmic burden, from 16% prior to surgery to 2% at 3 months, and less than 1% at 12 months.

**Figure 11:**
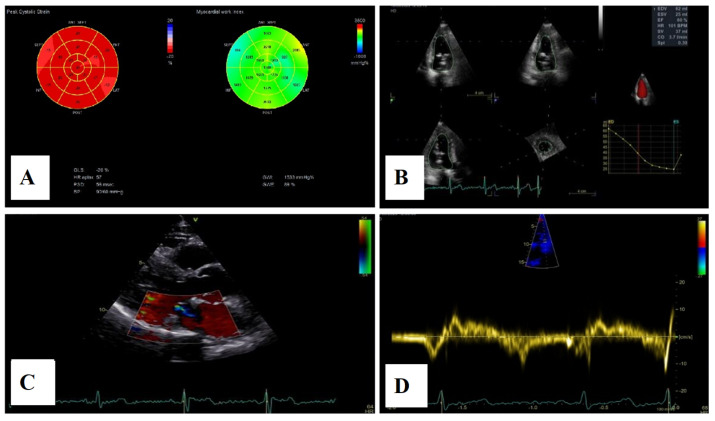
(**A**)—2D speckle-tracking echocardiography, displaying normal global longitudinal strain (−20%) and myocardial work bull’s-eye plot, displaying normal range of GWI (global work index—1533 mmHg%). (**B**)—Left ventricular volumetry and systolic function assessed by 3D echocardiography, showing normal volumes and ejection fraction—EF–60%. (**C**)—The parasternal long-axis view displays trivial mitral regurgitation without mitral annular disjunction. (**D**)—Tissue Doppler imaging on the lateral mitral annulus, without Pickelhaube sign.

Echocardiographic assessment demonstrated a reduction in left atrial and ventricular volumes, as measured by three-dimensional volumetry, with improvement in left atrial contractile function. Mitral regurgitation remained mild, and no recurrence of mitral annular disjunction was noted (Figure 11). Tricuspid regurgitation remained mild throughout the follow-up period, with a slight reduction in right atrial and ventricular dimensions and preserved right ventricular function.

Evaluation of the patient’s two daughters (aged 43 and 41) revealed the presence of mitral annular disjunction, without evidence of mitral valve prolapse, arrhythmias, or syncopal episodes.

## 3. Discussion

Mitral valve prolapse (MVP) is defined as one or both mitral leaflets billowing more than 2 mm above the annular plane during systole [5]. From a morphopathologic perspective, two primary etiologies are described: Barlow’s disease (myxomatous MVP) and fibroelastic deficiency [6,7,8,9,10,11,12].

MVP is the most prevalent valvular heart disease in the general population. According to the Framingham Heart Study, its prevalence is estimated at 1.3% for classic MVP and 1.1% for non-classic forms, with a slight female predominance [13,14,15]. Although most MVP cases are sporadic, familial clustering has been described, suggesting a possible genetic component. Further studies are needed to elucidate the underlying genetic determinants and assess the relevance of subclinical findings in first-degree relatives [16]. MVP is generally considered a benign condition, but clinical outcomes are highly variable, largely influenced by the presence and severity of mitral regurgitation and its sequelae [17,18,19,20,21,22,23]. Although the risk of sudden cardiac death and ventricular arrhythmias in patients with MVP—regardless of the degree of mitral regurgitation—was initially believed to be low, more recent observational studies suggest a higher incidence than previously thought. The estimated annual incidence ranges between 0.2% and 0.4% in the general MVP population, and may reach up to 1.8% in patients with severe mitral regurgitation [24,25,26].

**Mitral annular disjunction (MAD)** is defined as a systolic separation between the attachment of the left ventricular myocardium and the atrial wall–mitral valve junction, resulting in hypermobility of the mitral valve apparatus [27]. MAD has been described circumferentially around the mitral annulus, interspersed with normal tissue, and has also been observed at the level of the tricuspid annulus [28,29].

It can be identified in the parasternal long-axis view using transthoracic echocardiography or cardiac magnetic resonance imaging [30,31,32,33,34]. The width of the disjunction is measured in end-systole, from the insertion point of the posterior leaflet to the interface with the left ventricular myocardium, with reported values typically ranging from 5 to 10 mm [35]. Importantly, MAD has been associated with an increased risk of life-threatening ventricular arrhythmias, particularly when located along the posterior wall of the left ventricle [28,32,36].

However, current evidence is based largely on small and heterogeneous cohorts, with notable selection bias. There is no universally accepted definition of MAD regarding its annular extension or gap size, and it remains unclear whether it always represents a pathological finding or can sometimes be a normal variant. A modest reduction in arrhythmic risk has been observed following mitral valve surgery, particularly when annuloplasty is used to restore continuity between the mitral annulus and the left ventricular myocardium. This surgical correction appears to reduce mechanical stress on the papillary muscles, thereby removing a key trigger for ventricular arrhythmias—as illustrated in our patient, who showed a significant postoperative reduction in arrhythmic burden.

Both of the patient’s daughters were found to have mitral annular disjunction on echocardiography, but without mitral valve prolapse, hemodynamic compromise, arrhythmias, or symptoms. The clinical significance and optimal management of such findings remain uncertain. To date, there are no specific guidelines addressing the evaluation or follow-up of asymptomatic individuals with isolated MAD, making clinical decisions in such cases largely empirical. Unfortunately, our current understanding of MAD progression over time is limited, and further longitudinal studies are necessary.

**Arrhythmogenic mitral valve phenotype (AMVP)** is characterized by the presence of mitral valve prolapse (with or without associated mitral annular disjunction), accompanied by frequent and/or complex ventricular arrhythmias, in the absence of other identifiable arrhythmic substrates such as primary channelopathies, cardiomyopathies, myocardial scar, or active ischemia [37,38]. Large cohort studies have reported a female predominance among individuals with mitral valve prolapse who suffered sudden cardiac death [39,40]. A positive family history of sudden cardiac death represents a significant risk factor. Nevertheless, unexplained syncope is more frequently observed in patients with malignant arrhythmias [41,42] and may therefore serve as a clinical clue to high-risk AMVP.

Several mechanisms contribute to malignant ventricular arrhythmias in mitral valve prolapse, involving a complex interplay between anatomical substrates (regional hypertrophy, myocardial fibrosis, Purkinje system dysfunction), modulators (electrolyte disturbances, autonomic imbalance, altered hemodynamics), and triggers such as complex ventricular ectopy. Mechanical stretch of the papillary muscles may shorten the action potential duration and reduce resting diastolic potential, resulting in triggered activity [43]. In addition, repetitive mechanical stress may induce fibrotic changes in the papillary muscles and the endocardial and mid-myocardial layers of the left ventricle, creating abnormal repolarization gradients that manifest as inverted or biphasic T waves in the inferior and lateral leads on a 12-lead electrocardiogram [43,44]. In some patients, QT interval prolongation [45,46,47] and fragmented QRS complexes—indicative of localized myocardial scar—have also been observed [48,49].

The papillary muscles, the anterior and posterior fascicles, the outflow tract, and the mitral annulus are known sources of ventricular ectopy in AMVP and can serve as triggers for life-threatening arrhythmias [43,50]. Specific electrocardiographic markers have been proposed to help localize the origin of these arrhythmias, including QRS duration, r and R’ wave amplitude in lead V1, and precordial lead transition patterns [51,52,53]. Shorter QRS durations are associated with fascicular ectopy, while longer durations suggest a papillary muscle origin. Ectopy arising from the posterior annulus or postero-medial papillary muscle typically displays a superior axis, whereas ectopy from anterior structures tends to show an inferior axis. A r < R’ pattern in lead V1—resembling right bundle branch block—is characteristic of fascicular origin [51,54]. However, the prognostic value of the ectopic focus location remains uncertain [55].

The His–Purkinje conduction system may act as both trigger and substrate for arrhythmogenesis in AMVP, representing a critical component in the pathophysiology of ventricular arrhythmias and sudden cardiac death [56,57].

Routine screening with 24 h Holter monitoring is recommended for patients with mitral valve prolapse to assess arrhythmia burden, morphology, and coupling intervals. Patients with syncope and frequent or complex arrhythmias [58], polymorphic arrhythmias, short-coupled premature ventricular contractions (<350 ms) [59], or episodes of non-sustained or sustained tachycardia should undergo further investigation [24,58,60].

The patient’s ECG demonstrated T-wave inversion in leads DIII and aVF, reflecting repolarization abnormalities often observed in arrhythmogenic mitral valve prolapse, particularly in the infero-posterior region, which may correspond to localized myocardial stretch or fibrosis near the mitral annulus. Twenty-four-hour Holter monitoring revealed frequent polymorphic PVCs, with short-coupled beats, bigeminy, ventricular couplets, and episodes of non-sustained ventricular tachycardia. Based on established localization algorithms, these ectopic beats likely originated from the posterior and anterior papillary muscles and the mitral annulus, consistent with known arrhythmic substrates in MVP. The combination of repolarization changes, high PVC burden, and NSVT highlights the patient’s high arrhythmic risk.

Comprehensive echocardiographic assessment is crucial [4,61] and should include evaluation of mitral valve morphology and mitral annular diameter [62]. Mitral annular dilatation and flattening are frequently associated with mitral annular disjunction, which should be evaluated in terms of presence and length. A distinct echocardiographic feature—termed the “Pickelhaube sign”—described by Muthukumar et al. [63], represents a sharp mid-to-late systolic velocity spike at the lateral mitral annulus seen on tissue Doppler imaging, and has been proposed as a marker of arrhythmogenic mitral valve prolapse.

Quantification of mitral regurgitation and its hemodynamic impact is essential. Three-dimensional echocardiography has shown high agreement with cardiac magnetic resonance imaging in measuring left ventricular volume, ejection fraction, and regurgitant volume, particularly in cases with eccentric or multiple jets [5,64]. Assessment of myocardial deformation parameters such as global longitudinal strain and myocardial work may provide additional insights, although further validation is required.

In chronic severe mitral regurgitation due to mitral valve prolapse, volume overload leads to progressive left ventricular dilatation, eccentric hypertrophy, and increased wall stress, ultimately impairing systolic function. The presence of mitral annular disjunction may exacerbate this remodeling by enabling paradoxical systolic expansion of the posterior annulus (“curling”), focusing mechanical stress on the basal inferolateral wall and papillary muscles, and promoting focal myocardial fibrosis—a substrate for ventricular arrhythmias and reduced contractile reserve. For further structural characterization, assessment of the mechanism of mitral regurgitation, and risk stratification, cardiac magnetic resonance imaging should be performed in selected patients at experienced centers with 1.5 T or 3 T scanners [65,66,67,68]. Several studies have reported an association between non-ischemic late gadolinium enhancement (localized to the papillary muscles or patchy fibrosis in the inferior and basal left ventricle) and malignant arrhythmias [64,69,70]. The relevance of late gadolinium enhancement in other myocardial regions remains uncertain in this population.

In AMVP, the accepted multi-criteria decision approach is to integrate clinical history, ECG/ambulatory rhythm data, and multimodality imaging into a tiered risk-stratification pathway that then guides monitoring intensity and treatment [4,60,61,62,63,64,65,66,67,68,69,70,71] as outlined below:

Step 1—Identify the AMVP phenotype (clinical, ECG, imaging)

- Clinical: female sex, family history of sudden cardiac death, unexplained syncope or presyncope, palpitations [4].
- Resting ECG: T-wave inversion in the inferobasal leads, frequent, complex ventricular ectopy, QRS fragmentation.
- Echocardiography: severe myxomatous degeneration, bileaflet prolapse, mitral annular disjunction, Pickelhaube sign, severe mitral regurgitation, decreased left ventricular ejection fraction.
- Cardiac MRI: myocardial fibrosis, LGE in the inferobasal LV, papillary muscles, or peri-annular region.

Step 2—Quantify arrhythmia burden

- PVC burden: ≥5–10% or complex morphologies, NSVT (especially polymorphic, rapid, or short-coupled), or sustained VT/VF [4].
- If baseline features are high-risk but monitoring is negative, prolonged monitoring (implantable loop recorder (ILR)) is reasonable [4].

Step 3—Risk tiers that guide action

- **Lower risk:** MVP ± mild MR, no high-risk ECG features, no MAD or LGE, minimal ectopy requires periodic follow-up and lifestyle/risk-factor optimization.
- **Intermediate risk:** ≥1 phenotypic risk feature (bileaflet MVP, MAD, T-wave inversion, or focal LGE) without complex VT, consider intensified monitoring (24–72 h Holter or longer, consider ILR), consider β-blocker if symptomatic.
- **High risk:** Unexplained syncope, complex NSVT (fast/polymorphic/short-coupled), LGE, significant MAD with systolic curling, reduced LV function, or sustained VT/VF/cardiac arrest, consider catheter ablation for triggering PVCs/VT, ICD according to consensus-based criteria, and evaluate mitral surgery if severe MR or refractory arrhythmias related to valve-annular mechanics [4].

However, no single parameter—or combination of them—has yet been validated as a reliable predictor of sudden cardiac death in AMVP [60,71].

Contemporary mitral valve repair techniques, including leaflet resection or chordal replacement combined with annuloplasty, are effective in eliminating mitral regurgitation and restoring normal annular geometry. In patients with mitral annular disjunction, surgical repair typically eliminates the disjunction without the need for valve replacement.

**Reverse remodeling** refers to the regression of pathological cardiac chamber dilatation and hypertrophy, restoration of normal geometry, and stabilization or improvement of systolic function following resolution of volume or pressure overload. In patients with mitral valve prolapse, reverse remodeling occurs in distinct phases, as illustrated by the present case.

We reported the case of a 72-year-old female presenting with multiple syncopal and presyncopal episodes, a family history of sudden cardiac death, and complex ventricular ectopy on 24 h Holter monitoring. Echocardiography revealed severe mitral regurgitation due to bileaflet MVP with extensive myxomatous degeneration, mitral annular dilatation, MAD (10 mm), and the presence of the Pickelhaube sign. Although such findings are often associated with focal myocardial fibrosis on cardiac magnetic resonance imaging, no late gadolinium enhancement (LGE) was observed in this patient, suggesting the absence of detectable fibrosis.

**Figure 12:**
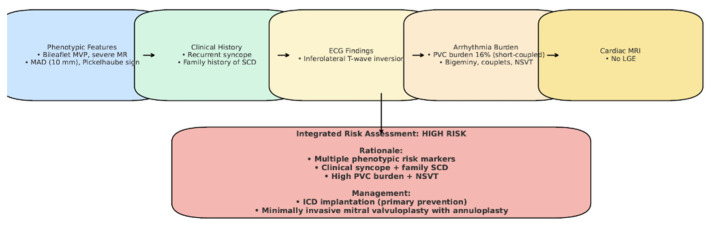
Case-specific schematic figure summarizing patient’s risk profile and management.

Although our patient did not exhibit sustained ventricular tachycardia, late gadolinium enhancement on cardiac MRI, or undergo electrophysiological testing, the decision to implant an ICD was based on the convergence of multiple high-risk features (Figure 12), in line with the 2022 EHRA Expert Consensus [4]. An electrophysiological study (EP study) was not performed, as the patient declined the procedure. Importantly, the 2022 EHRA consensus does not mandate EP testing in the risk stratification of AMVP, and ICD implantation may be considered in the presence of multiple high-risk features, even in its absence. After a multidisciplinary Heart Team evaluation, the patient was considered at elevated risk for sudden cardiac death (Figure 12). Furthermore, initiation of antiarrhythmic therapy with a beta-blocker was considered necessary to reduce the arrhythmia burden; however, given the patient’s baseline bradycardia (~55 bpm), pacing support was anticipated. This clinical context favored the implantation of a dual-chamber ICD rather than a single-chamber system. Taken together, the integration of phenotypic, arrhythmic, and clinical risk markers supported the decision for device therapy, despite the absence of sustained VT or LGE, as a proactive measure for primary prevention in a patient at high risk for sudden cardiac death. Beta-blocker therapy with bisoprolol was initiated to reduce arrhythmia burden.

To address the severe mitral regurgitation, the patient underwent three-dimensional video-assisted thoracoscopic mitral valvuloplasty and annuloplasty via a minimally invasive approach. Neochordae were implanted—four to the anterior leaflet and five to the posterior leaflet. This case supports the mechanical hypothesis for arrhythmogenesis in AMVP, suggesting that mitral valve surgery may contribute to the prevention of sudden cardiac death by reducing mechanical strain on the arrhythmic substrate [72,73,74,75,76,77,78,79]. However, further clinical trials are needed to clarify whether mitral surgery alone can sufficiently prevent ventricular arrhythmias in this population. Until more definitive data are available, ICD implantation remains a justified strategy in selected high-risk patients [4].

Postoperative echocardiographic assessment revealed a left ventricle of normal size and function, a hyperechogenic mitral annulus, and a structurally normal mitral valve without prolapse and only minor residual regurgitation. Two distinct groups of hyperechoic chordae were visualized at the level of the papillary muscles, confirming appropriate neochordae implantation.

Limitation: This report describes a single patient, which limits our findings. An electrophysiological study was not performed, as the patient declined the procedure. Similarly, genetic testing could not be carried out due to significant financial constraints in our country; the patient was informed of its potential value, but was unable to undergo testing. Additionally, while mitral annular disjunction (MAD) was observed, its significance in asymptomatic relatives remains unclear, and further studies are needed to clarify its predictive value for arrhythmic risk.

In summary, this case contributes to the evolving understanding of arrhythmogenic mitral valve prolapse by highlighting several unique aspects. First, long-term follow-up after surgical repair documented reverse remodeling and complete resolution of ventricular arrhythmias, emphasizing the importance of timely mitral intervention before irreversible myocardial damage, such as fibrosis, occurs. Second, our findings support the hypothesis that annuloplasty and restoration of annular integrity may stabilize or abolish MAD, thereby contributing to arrhythmia reduction. Finally, the familial context, with both daughters found to have MAD but no clinical manifestations, underscores the knowledge gap regarding whether MAD represents the earliest phenotypic marker and how such individuals should be followed over time. Together, these elements provide new insights into the interplay between MVP, MAD, ventricular arrhythmias, and the potential mechanistic role of surgical repair.

## 4. Conclusions

Although mitral valve prolapse without significant regurgitation is generally associated with a benign prognosis, identifying the subset of patients at risk for sudden cardiac death remains a clinical challenge, comparable to solving a puzzle with missing pieces. At present, no individual marker or combination of clinical, imaging, or electrocardiographic parameters has been validated to reliably predict long-term outcomes in these patients. Therefore, further clinical research is essential to develop and validate diagnostic tools, improve risk stratification, and establish evidence-based management strategies for patients with arrhythmogenic mitral valve phenotype.

Accurate identification of mitral annular disjunction has become increasingly important, as recent findings suggest its potential role as an arrhythmogenic substrate in patients with MVP. The association between MAD and ventricular arrhythmias, including frequent premature ventricular contractions and sudden cardiac death in patients without other apparent risk factors, highlights the need for comprehensive imaging assessment using advanced echocardiography and cardiac magnetic resonance techniques.

The familial occurrence of MAD, as illustrated in the present case, supports the hypothesis that this condition may have a hereditary component. Although genetic testing is a valuable tool in the evaluation of many cardiovascular disorders, its usefulness in MAD remains limited in the absence of specific genetic markers. Moreover, the familial finding of asymptomatic MAD highlights an important unanswered question—whether MAD is the earliest phenotypic marker of arrhythmogenic MVP and how such individuals should be monitored.

Surgical repair of the mitral valve in patients with MVP and MAD accomplishes two essential goals: correction of mitral regurgitation and restoration of annular continuity. This intervention contributes to reverse remodeling of the heart chambers, with reductions in left ventricular and atrial volumes, normalization of chamber geometry, preservation or improvement of systolic function, and possibly a decrease in arrhythmic burden. Early referral for surgery may enhance these outcomes, and targeted arrhythmia monitoring remains an important element in the long-term management of this patient population.

## Data Availability

No new data were created or analyzed in this study.
